# The functional importance of VCP to maintaining cellular protein homeostasis

**DOI:** 10.1042/BST20220648

**Published:** 2022-10-05

**Authors:** Brittany A. Ahlstedt, Rakesh Ganji, Malavika Raman

**Affiliations:** Department of Developmental Molecular and Chemical Biology, Tufts University School of Medicine, Boston, MA, U.S.A.

**Keywords:** autophagy, neurodegeneration, organelles, protein quality control, ubiquitin

## Abstract

The AAA-ATPase (ATPases associated with diverse cellular activities) valosin-containing protein (VCP), is essential for many cellular pathways including but not limited to endoplasmic reticulum-associated degradation (ERAD), DNA damage responses, and cell cycle regulation. VCP primarily identifies ubiquitylated proteins in these pathways and mediates their unfolding and degradation by the 26S proteasome. This review summarizes recent research on VCP that has uncovered surprising new ways that this ATPase is regulated, new aspects of recognition of substrates and novel pathways and substrates that utilize its activity.

## Introduction

**Figure 1.**
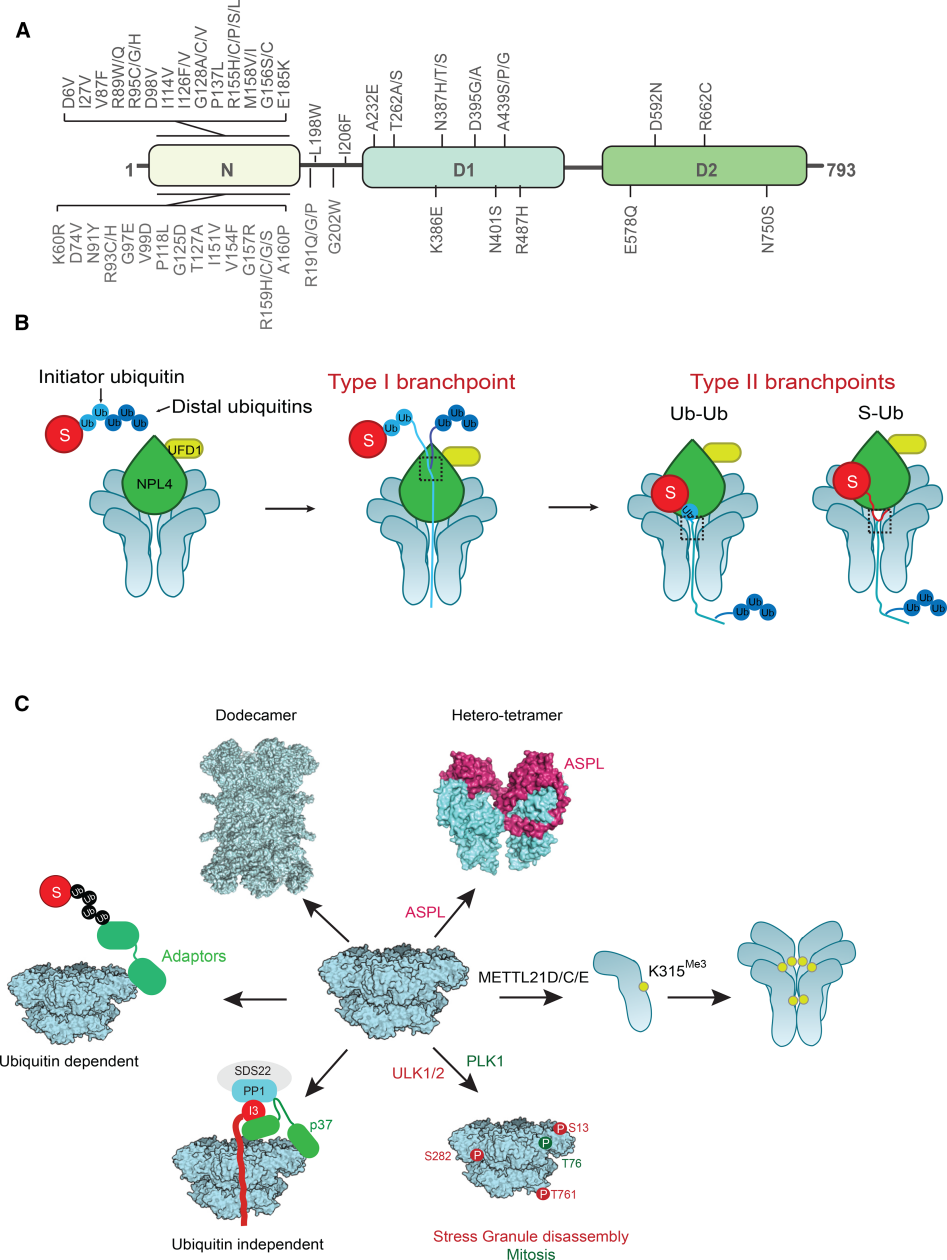
Structural organization of VCP and mechanism of substrate unfolding. (**A**) VCP protein structure. VCP assembles primarily as a homo-hexamer comprised of six monomers, each of which contains a globular N-domain and two ATPase domains (D1 and D2). Pathogenic mutations within each domain are indicated. (**B**) Directionality of substrate unfolding by Cdc48p. Cdc48p encounters a type I branch point when the initiator ubiquitin reaches the pore of the hexamer. Unfolding continues toward the C-terminus of the ubiquitin. At a type II branch point, Cdc48p can move toward the N- or C-terminus to eventually unfold the substrate. (**C**) Structure and substrate processing by VCP. VCP interacts with adaptor proteins to identify ubiquitylated substrates for degradation by the proteasome. Through interaction with p37, VCP can unfold substrates such as I3 independently of ubiquitylation. Phosphorylation of VCP by ULK1/2 increases the ATPase activity of VCP to enhance stress granule disassembly, while phosphorylation by PLK1 regulates VCP function in mitosis. Trimethylation of VCP monomers at Lys315 occurs by the action of METTL21D/C/E. The ASPL adaptor disassembles VCP hexamers to form ASPL–VCP hetero-tetramers (structure modeled in PyMOL using PDB 5IFS). Two hexamers that interact at the C-terminal tail provide an interface for the formation of dodecamers (structure modeled in PyMOL using PDB 7K57).

Valosin-containing protein (VCP, also p97, or Cdc48p in yeast) is an evolutionarily conserved, homo-hexameric, ubiquitin-selective, AAA-ATPase that functions in numerous ubiquitin-dependent protein quality control pathways. VCP identifies ubiquitylated substrates through numerous dedicated adaptor proteins and unfolds substrates by threading them through a central pore in the hexamer (Figure 1). Due to its abundance and versatile function, VCP participates in many cellular pathways including ERAD, endolysosomal trafficking, selective autophagy, cell cycle regulation, and DNA damage signaling [1]. We recommend several excellent reviews that discuss these well-established functions [1–4].

In this review, we discuss new research that illuminates how VCP processes ubiquitylated substrates, surprising roles for VCP in ubiquitin-independent unfolding and new pathways that rely on VCP activity. We also review current findings related to how VCP mutations contribute to human degenerative disorders.

## Structural insights into substrate processing by VCP

VCP functions primarily as a homo-hexamer wherein each monomer contains an N-terminal domain followed by two ATPase domains (D1 and D2) (Figure 1A) [1]. Depending on the nucleotide bound state of the hexamer, the N-domain exhibits either a co-axial conformation (‘up’) or a co-planar position (‘down’) with respect to the D1–D2 ring. While both D1 and D2 ATPase domains are functional, ATP binding in the D1 domain promotes hexamer stability whereas ATP binding and hydrolysis by the D2 domain generates the force necessary for unfolding [5,6]. The mechanism by which VCP processes substrates has been intensely debated in the field. Recent cryo-electron microscopy (cryo-EM) structures of mammalian VCP or its yeast ortholog Cdc48p bound to distinct adaptors (the UFD1–NPL4 [U–N] dimer or p47 [Shp1p in yeast]), have now shed light on this mechanism [7–10]. Consecutive ATP hydrolysis by each monomer enables VCP to unfold associated substrates by pulling them through the central channel of the hexamer [7,9]. ATPase activity is coupled to unfoldase activity providing a nucleotide-dependent regulation of substrate unfolding [5,6]. Unfolded substrates are subsequently degraded by the proteasome. UFD1 binds ∼2–3 ubiquitin molecules via its UFD1 truncation-3 (UT3) domain and NPL4 binds ∼3 ubiquitin molecules via the C-terminal MPR1/PAD1-N-terminal (MPN) domain. Thus, the VCP–U–N complex can accommodate ∼5–6 ubiquitin molecules distributed between VCP and U–N (Figure 1B) [7–9,11]. NPL4 forms a tower over the VCP hexamer pore and binds to two folded ubiquitins followed by one unfolded ubiquitin that binds to a groove in NPL4 such that it is positioned near the entry to the D1 ring of Cdc48p. This unfolded ubiquitin serves as the initiator ubiquitin (Figure 1B) [7–9,11]. The choice of the initiator ubiquitin appears to be random, however, since these studies only used substrates with five ubiquitin moieties, whether there is a preference when longer chains are present remains to be elucidated. The free N-terminus of this initiator ubiquitin molecule is partially unfolded due to interactions with a series of conserved residues in the NPL4 groove [11]. The partially unfolded N-terminus of the initiator ubiquitin is inserted into the D1 pore loop in an ATP hydrolysis-independent manner followed by the hand-over of the initiator ubiquitin to the D2 pore loop (Figure 1B). This hand-over is facilitated by the lower binding affinity of D1 pore loop residues for unfolded peptides compared with high-affinity aromatic residues in the D2 pore loop. As D2 pore loop residues pull on the ubiquitin to unfold it in an ATP hydrolysis-dependent manner, the terminal protomer in the hexamer moves out of hexamer symmetry to initiate a hand-over-hand or spiral-staircase motion observed in other AAA-ATPases [7–9,11]. Residues that occupy the inter-subunit interface between D1 and D2 (Leu335 in D1 and Met611 in D2 domains) interact with hydrophobic residues in Walker B motifs of neighboring subunits, further strengthening the interaction with ATP which is required for the spiral-staircase movement [12]. A similar mechanism was confirmed for the human VCP–p47 complex [10]. Unlike Cdc48p, the ATPase activity of the D1 and D2 domains in human VCP is asynchronous and the D1 and D2 domains that detach from hexamer symmetry are not from the same protomer [12]. N- or C-terminal tail truncations of human VCP have lower unfoldase activity suggesting their involvement in inter-subunit communication and spiral-staircase movement [12].A recent report by the Rapoport group further illuminates the directionality of substrate unfolding by Cdc48p [11]. During the unfolding of polyubiquitylated substrates, Cdc48p encounters two types of branch points termed type I and II (Figure 1B). The so-called type I branch point is the linkage between the Lys48 residue of initiator ubiquitin and C-terminus of distal ubiquitin (i.e. the ubiquitin moiety distal to the initiator ubiquitin) which is encountered when the initiator ubiquitin enters the hexamer pore (Figure 1B). Here, Cdc48p prefers unfolding in the direction of the C-terminus of unfolded initiator ubiquitin instead of towards distal ubiquitin. Unfolding continues until Cdc48p encounters the type II branchpoint which is either the lysine residue of the substrate or a Lys48 of another ubiquitin. At this branch point, Cdc48p can move towards either the N- or C-terminus to eventually unfold the substrate (Figure 1B) [11]. It is unclear how the distal folded ubiquitins traverse from the N-terminus to C-terminus of Cdc48p. One possibility is that the distal ubiquitin chain slides through the spaces between the protomers in the hexamer as asymmetry is broken during unfolding. However, the exact mechanism, and the specific residues involved in this process remain to be elucidated. These studies primarily utilize Lys48-linked ubiquitin chains. However, in cells VCP complexes associate with a diverse array of ubiquitin linkages including branched ubiquitin chains. Ubiquitin remnant profiling in cell treated with VCP small molecule inhibitors, NMS873 or CB5083, found an increased abundance of proteins carrying Lys6, Lys11, Lys48, Lys11/Lys48, and to a lesser extent Lys63 ubiquitin linkages [13]. This suggests that VCP may have relative specificity towards certain ubiquitin linkages on the substrate, which will determine the fate of the substrate. Very little is known about the ubiquitin linkage specificity of VCP adaptors. Future studies aimed at determining whether adaptors have preferential binding to certain ubiquitin linkages is required.

Intriguingly, other oligomeric states of VCP have also been identified. The alveolar soft part sarcoma locus (ASPL) adaptor can bind and disassemble VCP hexamers to form ASPL–VCP hetero-tetramers (Figure 1C) [14]. Although the physiological function of this assembly is unclear, it has been observed that overexpression of ASPL results in inhibition of VCP ATPase activity, perturbing ERAD, and subsequently causing cell death. A cell-permeable polypeptide derived from the ASPL UBX domain causes cancer cell death suggesting a yet unknown physiological role of VCP:ASPL hetero-tetramers [15]. Two VCP hexamers that assemble in a tail-to-tail fashion (dodecamers) in a nucleotide-free state have also been observed *in vitro* (Figure 1C) [16–22]. The C-terminal tail provides an interface for interactions between the D2 rings of the hexamers. Interestingly, cryo-EM studies using VCP Arg155His, a disease-relevant mutant, found that VCP mutants have an increased propensity to form dodecamers relative to wildtype [22]. Because nucleotide-free VCP dodecamers have not been observed *in vivo*, it is not clear if this state exists physiologically. Moreover, the functions of these hetero-tetrameric and dodecameric structures are not known. One possible role that has been proposed is that these structures may help sequester VCP unfoldase activity. Interestingly, several VCP adaptors including the E4 ubiquitin ligase ubiquitin fusion degradation 2 (UFD2) and UFD3 bind the C-terminus of Cdc48p. These adaptors enable further polyubiquitylation or aid in the deubiquitylation of substrates, respectively [1,23,24]. Hence, the sequestration of the C-terminal tail by dodecamer formation may disrupt the interaction between adaptors that interact with this region of Cdc48p. How dodecamers impact these interactions and the impact on downstream pathways remains to be elucidated.

Together, these structural studies have enabled us to understand the mechanism of substrate unfolding by VCP and its adaptors. However, whether and how these different VCP ensembles are built and disassembled in cells is a mystery and required further study.

## Ubiquitin-independent substrate processing by VCP

VCP is primarily a ubiquitin-dependent molecular machine, but recent studies have demonstrated that ubiquitin is not essential for VCP-mediated unfolding. Protein phosphatase-1 (PP1) is one of the major protein phosphatases in eukaryotes and facilitates numerous dephosphorylation reactions by associating with distinct regulatory subunits [25]. PP1 is held in an inactive complex by association with SDS22 and inhibitor-3 (I3) and must be disassembled for PP1 to interact with its regulatory subunits that target it to specific substrates [26]. Research from the Meyer group has reported that VCP in complex with the SEP domain-containing adaptor p37 dissociates the inactive PP1 complex by unfolding I3 through the central pore of the hexamer (Figure 1C) [27,28]. Surprisingly, this process does not require I3 to be ubiquitylated. SEP domain-containing adaptors such as p37 utilize a dual recognition mechanism to dissociate the complex. A hydrophobic region within the amphipathic helix of the SEP domain binds to an internal recognition site within I3 and directs this loop to the central pore of the hexamer [27,29]. PP1 is precluded from unfolding and held clear of the VCP pore by the linker between the SHP box and UBX domain in p37 [27,29]. The identification of the internal recognition site in I3 is interesting as it suggests that a free terminus is not required for I3 unfolding. Indeed, a circularized version of I3 is unfolded analogously to linearized I3 [29,30]. This is unexpected in two ways. First, the unfolding of ubiquitylated substrates is initiated by the U–N complex which identifies and inserts the free N-terminus of a ubiquitin molecule into the central pore of VCP [7,9,31]. Second, the ability of VCP to unfold a circularized I3 suggests that two polypeptides may occupy the VCP pore at once. This finding suggests that the pore of VCP may be flexible to accommodate a larger array of substrates, but this remains to be demonstrated. A recent cryo-EM structure of the VCP:p37:PP1 complex further sheds light on this process. This report, while confirming the spiral-staircase model, made an interesting observation that I3 can make direct contact with the N-domain of VCP in contrast with previous findings that adaptors act as mediators between VCP and the substrate [30]. While further high-resolution studies are required to gain insight into this mechanism, these findings raise the intriguing possibility that VCP may target more unmodified substrates and have a greater substrate repertoire than currently appreciated.

## Regulation of VCP function by post-translational modifications

**Figure 2.**
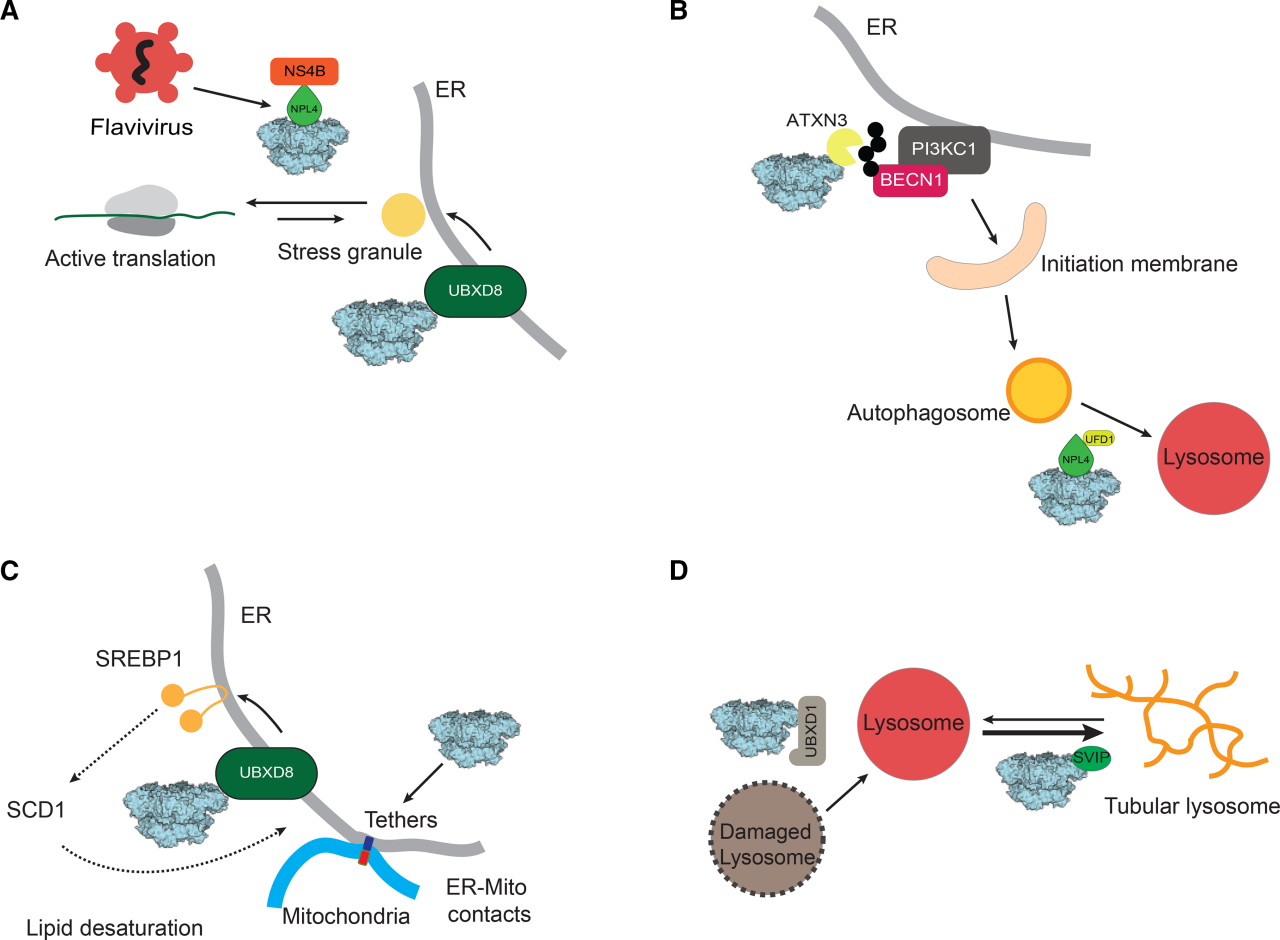
Novel VCP-dependent cellular functions. (**A**) VCP is hijacked by flaviviruses to disassemble stress granules to free the viral replication machinery through a direct interaction between the NPL4 adaptor and viral protein, NS4B. VCP–UBXD8 also regulates stress granule disassembly at the ER. (**B**) The interaction between VCP and ataxin-3 stimulates the deubiquitylation of the Ptdlns3K complex component Beclin1, stabilizing the complex and initiating phagophore formation. (**C**) The VCP–UBXD8 complex regulates ER-mitochondria contact sites by maintaining SCD1 protein levels and thereby membrane lipid saturation and composition. VCP an also directly modulate tethering proteins. (**D**) VCP plays multiple roles in several steps of autophagy. VCP–U–N plays a role in autophagosome-lysosome fusion, and VCP–UBXD1 functions in the clearance of damaged lysosomes (lysophagy). The VCP–SVIP complex maintains the integrity of the tubular lysosome network.

Several studies have revealed that phosphorylation and methylation of VCP influence its activity in numerous disease-relevant pathways (Figure 1C). Stress granules are membrane-less condensates that contain pre-initiation complexes, mRNAs and RNA binding proteins and arise in response to various stressors. VCP is recruited to stress granules via the ER tethered adaptor UBXD8 bound to the ubiquitylated stress granule protein G3BP1 (Figure 2A) [32]. The autophagy-inducing kinases ULK1 and ULK2 form a stress-induced complex with VCP and phosphorylate VCP at multiple sites (Ser13, Ser282, Thr761). Phosphorylation of these sites causes an increase in VCP ATPase activity and enhances the ability of VCP to disassemble stress granules [33]. Perturbed stress granule disassembly is linked to the pathogenesis of multisystem proteinopathy 1 (MSP-1) caused by VCP mutation. However, it is unknown if phosphorylation of these sites is impaired in VCP mutants known to cause disease.

Phosphorylation of the N-domain of VCP on Thr76 by polo-like kinase (Plk1) recruits VCP to the centrosome and regulates proper centrosome orientation during M-phase (Figure 1C). Dephosphorylation of Thr76 by the phosphatase PTEN at the centrosome relocates VCP to the spindle to ensure proper spindle structure and chromosome segregation [34]. Highlighting the importance of this phosphorylation, VCP Thr76Ala was unable to localize to either the centrosome or spindle [34]. These cells had chromosome defects and a significant delay in chromosome segregation [34]. Furthermore, mass-spectrometry studies identified kinesin 5 (Eg5) as a VCP interactor that was dependent on phospho-Thr76 for interaction [34]. Eg5 protein abundance and total ubiquitin levels do not change when VCP is phosphorylated, suggesting that phosphorylated VCP acts as a scaffold that is essential for Eg5 recruitment to the spindle [34].Phosphorylation of VCP at specific sites has been hypothesized to impact its activity in different ways (Figure 1C) [1]. Ser282 is located above the central pore of VCP, and phosphorylation of this site may enhance binding to the U–N complex [5,33,35]. Thr761 is located at the C-terminal end of the D2 domain [5]. An ionic bond between this phosphorylated residue and a residue on an adjacent protomer may contribute to the increase in ATPase activity [33,36]. Thr76 phosphorylation is hypothesized to strengthen inter-domain interaction between the N-domain and D1 domain by enhancing the Thr76–Arg83 hydrogen bond [34]. This influences the conformation of the nucleotide-binding pocket to better accommodate nucleotide binding [34].

Recent studies have identified a novel family of lysine methyltransferases that participate in protein quality control pathways by methylating molecular chaperones [37]. Class I methyltransferases METTL21C, D and E trimethylate VCP at Lys315 (Figure 1C) [37–41]. Curiously, as this site is buried deep in the hexamer and is not solvent exposed, it is thought that trimethylation occurs exclusively on unassembled VCP monomers. Notably, multiple studies utilizing blue native-PAGE and *in vitro* methylation assays have demonstrated that trimethylation boosted VCP complex assembly, but the methyltransferases could not trimethylate an already intact hexamer [38,39,41]. Whether methylated hexamers have altered function is unknown. Or perhaps more tantalizingly, are there roles for methylated monomers in the cell? Trimethylation of VCP is enhanced by ASPL, which was shown to interact directly with METTL21D by tandem affinity purification [37]. Whether trimethylation occurs exclusively after ASPL-mediated hexamer disassembly or if it also happens on newly translated monomers prior to hexamer formation is unclear. The enhanced methylation of VCP by ASPL and METT21D was abrogated in the presence of the Arg155His disease mutation which disrupts N-domain adaptor binding [37].

It appears that METTL21C and E only function in the skeletal muscle of mice, whereas METTL21D appears to function in diverse tissue types [37–41]. More studies are needed to parse out the exact function of VCP trimethylation. Of note, METTL21D knockout mice develop normally with comparable growth and proliferation rates to wild-type mice, even though VCP is not methylated in this model [39]. Thus, further studies are needed to fully elucidate how methylation of VCP impacts its cellular function.

## The role of VCP in viral infection

VCP facilitates the propagation of various positive-stranded RNA viruses including those of the *Flaviviridae* and *Coronaviridae* family [42–47]. VCP participates in multiple stages of viral production including viral entry, uncoating, intracellular trafficking, replication, and egress [42]. Extensive studies have demonstrated that viruses often hijack VCP-mediated ERAD to escape the immune system and maintain their proliferation. Early work from the Ploegh laboratory determined that the human cytomegalovirus (HCMV) proteins US11 and US2 enable the removal of newly synthesized major histocompatibility complex (MHC) class I molecules from the ER to the cytosol, resulting in the proteasomal degradation of class I heavy chains and enabling evasion from the immune system [48,49]. A recent study found that at late stages post-infection, cytomegalovirus-M50 protein uses ERAD to enable degradation of the ER-resident stress sensor, IRE1 thus attenuating the unfolded protein response. To prevent cell death and reduced viral titers, cytomegalovirus-M50 tethers IRE1 to SEL1L, an adaptor to the HRD1 E3 ligase, so it is recognized as an ERAD target and degraded through VCP and the proteasome [50].

Flaviviruses like dengue fever and Japanese encephalitis virus also hijack VCP and ERAD to degrade ubiquitylated non-structural viral proteins to maintain viral protein homeostasis (Figure 2). Recent studies illustrate a direct interaction between NPL4 and the viral protein NS4B which recruits VCP to sites of viral replication to disassemble stress granules and free viral replication machinery (Figure 2A) [51]. In this way, flaviviruses ensure that replication continues regardless of stress-induced translation inhibition [51]. Additionally, considerable evidence shows that VCP is vital for the escape of many viruses from organellar compartments like the ER and endosomes during infection. For example, multiple studies have demonstrated that VCP-dependent ERAD is co-opted for cholera toxin escape from the ER [52–56]. VCP has also been implicated in assisting early stages of coronaviral escape from endosomes into the cytosol to initiate viral replication [57]. Recently, a study utilizing quantitative proteomics found that coronaviruses utilize VCP to co-opt the host cell cycle to ensure viral survival [46]. Collectively, these studies indicate that VCP is a key cellular node targeted by viruses during infection and suggest that VCP is an attractive therapeutic target for the development of new antiviral compounds. Multiple ATP-competitive, covalent, and allosteric VCP inhibitors have been developed as anti-cancer compounds and may be re-purposed or further developed as anti-virals [42,44,58–60].

## New roles for VCP in autophagy

VCP has been implicated in the autophagic clearance of ubiquitylated protein aggregates, damaged lysosomes, and mitochondria. Early work found that VCP Arg155His and Ala232Glu disease mutants impair autophagosome maturation and fusion with lysosomes (Figure 2B) [61–64]. Recent studies have identified a new role of VCP in the early stages of autophagy initiation [65]. VCP in association with the deubiquitylase ataxin-3 interacts with Beclin1, a key component of phosphatidylinositol-3-kinase (PI3K) complex I. The PI3K complex I is required for phosphatidylinositol-3-phosphate [PI(3)P] synthesis on initiation membranes, a key step for the recruitment of downstream autophagy factors. Binding to VCP increases ataxin-3 deubiquitylase activity resulting in Beclin1 stabilization (Figure 2B). *In vitro* reconstitution studies indicate that VCP also promotes the assembly of Beclin1–PI3K complex I resulting in increased kinase activity and production of PI(3)P [65]. Taken together these studies suggest that VCP has unique roles in multiple, distinct steps of autophagy.

## Regulation of ER-Mitochondrial membrane contact sites

Inter-organellar communication via membrane contact sites between the ER and mitochondria plays an essential role in lipid metabolism, ion transport and autophagy [66,67]. Mutations in several ER-mitochondria contact site proteins have been associated with neurodegenerative diseases including amyotrophic lateral sclerosis (ALS), Alzheimer's, and Parkinson's diseases suggesting that perturbation in contacts and resident functions can contribute to disease pathology [68]. Multiple reports indicate that VCP and select adaptors may regulate the contact site proteome to modulate its function. VCP dissociates ER-mitochondria contacts by extracting and facilitating proteasomal degradation of contact tethers mitofusins 1 and 2 to initiate mitophagy [69]. VCP has also been shown to interact with VPS13D to regulate the abundance of the contact site tether protein, VAPB [70]. However, the mechanism by which this occurs is not known. VCP and the ERAD adaptor, UBXD8 regulate ER-mitochondria contacts by regulating membrane lipid saturation and composition (Figure 2C) [71]. VCP–UBXD8 regulates contacts by modulating the expression of the fatty acid desaturase SCD1 through the SREBP1 pathway. VCP–UBXD8 mediate the ubiquitin-dependent degradation of INSIG1, which holds the SREBP1 transcription factor in an inactive trimeric complex along with SCAP1 at the ER. INSIG1 degradation activates SREBP1 processing, freeing the transcription factor to translocate to the nucleus to activate numerous genes involved in lipid metabolism including SCD1. Loss of VCP–UBXD8 stabilizes INSIG1, inactivating SREBP1, and decreasing SCD1 transcript and protein levels. This, in turn, leads to an increase in membrane saturation thereby stabilizing contacts (Figure 2C) [71]. This new function of VCP appears to be relevant in the context of disease. Motor neurons derived from ALS patients carrying the VCP Arg155His mutation have an increase in ER-mitochondria contacts and mouse models of VCP disease display a decrease in SREBP1 processing and depleted SCD1 protein levels in the brain [71,72]. Whether VCP can regulate other organelle contacts is not known but the loss of SCD1 protein and altered membrane saturation has been shown to impact ER-plasma membrane contacts [71].

## The role of VCP in tubular lysosome function

Lysosomes are essential cellular quality control organelles that are often depicted as vesicular structures. In some biological contexts, lysosomal membranes have been found to expand into extended tubular structures, forming a network that spreads throughout the cell [73]. Elegant live imaging studies in *Drosophila* expressing the lysosomal transmembrane protein Spinster, have revealed a dynamic network of tubular lysosomes in the muscle that undergo constant extension, breakage, and merging [74]. VCP has been found to play an essential role in the maintenance of this network and concentrates on tubular lysosomes (Figure 2D). Overexpression, genetic depletion, or pharmacological inhibition by the VCP inhibitor, DBeQ resulted in the collapse of lysosome tubules into vesicular structures that were no longer dynamic [74]. Disruption of the structural properties of the tubules impaired autophagosome–lysosome fusion, resulting in increased cytoplasmic ubiquitylated aggregates, damaged mitochondria, and impaired muscle function [74]. Importantly, disease-causing mutations in VCP perturb tubular lysosomes, resulting in the accumulation of cytoplasmic lipid granules [74].

More recently, the adaptor small VCP-interacting protein (SVIP) was identified to recruit VCP to tubular lysosomes to maintain their integrity (Figure 2D). SVIP knockout (KO) in flies phenocopies VCP depletion by impairing autophagosome-lysosome fusion, destabilizing tubules, and accumulating insoluble aggregates [75]. SVIP KO *Drosophila* demonstrate motility defects mirroring progressive decline in the motor system [75]. VCP disease-relevant mutations in the N-domain severely impact SVIP binding and prevent VCP recruitment to tubular lysosomes [75]. Notably, a mutation in SVIP (Ser77Leu) was found in a patient with sporadic fronto-temporal dementia [75]. SVIP Ser77Leu mutants were found to disrupt tubular lysosomes and cause muscle degeneration in flies [75].

While tubular lysosomes are essential for muscle cell viability, it remains elusive why this network is necessary. Many questions remain such as what drives lysosomal membranes to form tubules and what proteins regulate their fission and fusion. Additionally, the mechanistic role of VCP in either initiation or preservation of this network remains to be elucidated. Answers to these questions will likely provide insight into how dysfunction of this network is implicated in degenerative disease.

## Aberrant VCP regulation is linked to multiple human diseases

More than 45 missense mutations in VCP have been identified in multiple neurodegenerative diseases (Figure 1A). These include familial ALS, Parkinson's disease, Charcot Marie Type-2B, vacuolar tauopathy and inclusion body myopathy, Paget's disease, and fronto-temporal dementia (IBMPFD) also known as MSP-1 [76–79]. Protein aggregates containing alpha-synuclein, Tau, or Tar-DNA binding protein 43 (TDP43) are frequently observed in VCP-associated neurodegenerative disorders [76–79]. Mutations in VCP compromise its function in various cellular protein quality control pathways resulting in the accumulation of aggregated and ubiquitylated proteins, ER stress, mitochondrial dysfunction, and cell death [1,76,77]. Recent *in vitro* reconstitution studies of disease-linked VCP mutants suggest a toxic gain-of-function phenotype with increased ATPase and unfoldase rates wherein the ATPase activity is uncoupled from unfoldase activity [80,81]. While it is unclear how increased unfoldase activity perturbs VCP-dependent pathways, one possibility is that the 26S proteasome cannot keep up with the increased rate of substrate unfolding leading to aberrant substrate accumulation.

Many disease mutations in VCP accumulate in the N-D1 linker region leading to an activated conformation (Figure 1A) [76]. This conformation perturbs specific adaptor interactions thus inhibiting downstream processes [1,82–85]. For instance, the VCP Arg155His has decreased affinity for UBXD1 but increased interactions with U–N, and p47. Thus, the role of VCP–UBXD1 in endo-lysosome trafficking and autophagy of damaged lysosomes (lysophagy), is impaired by VCP mutation (Figure 2) [83]. Indeed, ALS tissue biopsies from patients with the Arg155His mutation have aberrant accumulation of damaged lysosomes [86]. The U–N dimer is involved in most VCP-dependent processes including ERAD and increased binding may contribute to defects in these pathways. The VCP Arg155His mutant has been recently reported to increase the seeding of alpha-synuclein and TDP43 aggregates, likely due to decreased UBXD1 binding and loss of endolysosomal integrity [87–90].

A VCP Asp395Gly hypomorph mutation has been reported to cause vacuolar tauopathy, an autosomal-dominant form of dementia [87]. The VCP Asp395Gly mutant had lower ATPase activity and was unable to unfold and clear Tau aggregates suggesting a loss-of-function phenotype [87]. Taken together, VCP disease mutations are implicated in both gain-of- and loss-of-function phenotypes. Together with the diverse disease phenotypes observed in patients and the multitude of substrates that VCP regulates, it has been a challenge in the field to identify points of intervention for disease therapy. At the recent Cure VCP Disease conference, the need for standardized assays and relevant *in vitro* cell and animal models to systematically assess how distinct VCP mutations impact specific phenotypes were highlighted as an unmet need [91].

Increased VCP protein expression is observed in many cancers and correlates with poor patient outcomes. Hence, targeting VCP activity has emerged as a promising therapeutic option in cancer. Several allosteric and ATP-competitive small molecule inhibitors have been developed and have been shown to have anti-tumor activity in pre-clinical models [92]. These inhibitors activate ER stress and induce apoptosis in cancer cells. Based on these observations, a phase I clinical trial was initiated with the VCP ATP-competitive inhibitor, CB5083 for the treatment of solid tumors and multiple myeloma [93]. However, due to potent off-target effects on the vision of the patients, the clinical trial was discontinued [94,95]. Prolonged exposure to CB5083 resulted in the acquisition of resistance in several cancer cell lines due to the emergence of mutations in *VCP*. The mutations that render resistance to CB5083 are in D1–D2 linker region or in D2 ATPase domain thus altering either intra-subunit D1–D2 communication or blocking the binding pocket of CB5083 [96–101]. Nevertheless, these barriers have been overcome in the development of the next-generation inhibitor, CB5339. Phase I clinical trials using CB5339 are ongoing to study its potency against solid tumors and acute myeloid leukemia (AML) or myelodysplastic syndrome [95]. Because some MSP-1-linked mutations in VCP are associated with increased ATPase activity, recent studies have explored whether small molecule inhibitors of VCP can alleviate MSP-1-associated phenotypes. Indeed, initial reports support the idea that CB-5083 can alleviate some of the cellular defects associated with VCP mutation [94,102]. These findings have spurred studies to investigate whether CB5083 can be re-purposed towards treating VCP-associated myopathy [94]. Given the pleiotropic nature of MSP-1 and the numerous mutations associated with this disorder, it is not clear how effective the current inhibitors will be in the mitigation of disease phenotypes.

## Concluding remarks

The VCP research community has made great strides in elucidating the molecular mechanisms and pathways orchestrated by VCP. Many of these processes have been validated to be perturbed in diseases caused by VCP mutations. However, we continue to be surprised by new ways in which VCP recognizes and processes its substrates; no doubt there are many more substrates and pathways that remain to be uncovered. The looming challenge is to ask which of these functions are relevant to VCP disease? With so many mutations that are reported to perturb function in distinct ways and diverse patient symptoms, how can we develop therapeutic compounds that can modulate disease? A concerted effort to develop standardized assays that monitor VCP function in distinct pathways, systematic assessment of substrates in a cell or tissue-specific manner, and cell or animal models that recapitulate various aspects of the disease are critically needed.

## Perspectives

- VCP is an essential protein involved in a multitude of protein quality control events to maintain protein homeostasis. Aberrant VCP regulation and function can cause cancer as well as several neurodegenerative diseases.
- VCP is primarily considered a ubiquitin-dependent, homo-hexameric molecular chaperone, but recent studies have illustrated the diversity of VCP structures, new aspects of substrate identification and novel cellular pathways.
- These studies highlight the diversity of VCP function and the need to systematically determine how these processes are disrupted in VCP-related disorders.

## Abbreviations

ALS: amyotrophic lateral sclerosis
ASPL: alveolar soft part sarcoma locus
cryo-EM: cryo-electron microscopy
ERAD: endoplasmic reticulum-associated degradation
PI3K: phosphatidylinositol-3-kinase
PP1: protein phosphatase-1
TDP43: Tar-DNA binding protein 43
VCP: valosin-containing protein
